# Manual and semiautomatic segmentation of bone sarcomas on MRI have high similarity

**DOI:** 10.1590/1414-431X20198962

**Published:** 2020-01-31

**Authors:** F.C.F. Dionísio, L.S. Oliveira, M.A. Hernandes, E.E. Engel, R.M. Rangayyan, P.M. Azevedo-Marques, M.H. Nogueira-Barbosa

**Affiliations:** 1 Departamento de Imagens Médicas, Hematologia e Oncologia Clínica, Faculdade de Medicina de Ribeirão Preto, Universidade de São Paulo, Ribeirão Preto, SP, Brasil; 2 Laboratório de Pesquisa em Imagens Musculoesqueléticas, Faculdade de Medicina de Ribeirão Preto, Universidade de São Paulo, Ribeirão Preto, SP, Brasil; 3 Department of Electrical and Computer Engineering, University of Calgary, Calgary, Alberta, Canada

**Keywords:** Ewing sarcoma, Osteosarcoma, Manual segmentation, Semiautomatic segmentation

## Abstract

The aims of this study were to evaluate the intra- and interobserver reproducibility of manual segmentation of bone sarcomas in magnetic resonance imaging (MRI) studies and to compare manual and semiautomatic segmentation methods. This retrospective study included twelve osteosarcoma and eight Ewing sarcoma MRI studies performed prior to any therapeutic intervention. All cases were histopathologically confirmed. Three radiologists used 3D-Slicer software to perform manual segmentation of bone sarcomas in a blinded and independent manner. One radiologist segmented manually and also performed semiautomatic segmentation with the GrowCut tool. Segmentation exercises were timed for comparison. The dice similarity coefficient (DSC) and Hausdorff distance (HD) were used to evaluate similarity between the segmentation results and further statistical analyses were performed to compare DSC, HD, and volumetric results. Manual segmentation was reproducible with intraobserver DSC varying from 0.83 to 0.97 and HD from 3.37 to 28.73 mm. Interobserver DSC of manual segmentation showed variation from 0.73 to 0.97 and HD from 3.93 to 33.40 mm. Semiautomatic segmentation compared to manual segmentation resulted in DSCs of 0.71−0.96 and HDs of 5.38−31.54 mm. Semiautomatic segmentation required significantly less time compared to manual segmentation (P value ≤0.05). Among all situations compared, tumor volumetry did not show significant statistical differences (P value >0.05). We found excellent intra- and interobserver agreement for manual segmentation of osteosarcoma and Ewing sarcoma. There was high similarity between manual and semiautomatic segmentation, with a significant reduction of segmentation time using the semiautomatic method.

## Introduction

Primary bone sarcomas are rare and represent about 0.2% of all malignant tumors in the general population (1), but about 5% of all primary malignant neoplasms in the pediatric population, with osteosarcoma and Ewing sarcoma being the most frequent in this group (2,3). Magnetic resonance imaging (MRI) is considered the best method for regional staging of primary bone sarcoma (3) because it facilitates the evaluation of several tumor features, such as anatomical extension of the tumor, compromise of the growth plate, affected neurovascular or articular structures, and the presence of skip lesions (3,4). Primary bone sarcomas still have significant morbidity and mortality rates (3). In this context, new research is important to develop new diagnostic strategies and therapeutic modalities aiming, for example, to individualize the treatment scheme to obtain better outcomes.

Radiomics has emerged as a process of extracting measurements and attributes from medical images that can generate relevant information for clinical decisions. The steps related to radiomics include the extraction of quantitative data from areas of interest in medical images, the storage of these data, and their use to generate and test clinical hypotheses (5). To extract data from medical images, the segmentation of areas or volumes of interest is usually necessary. There are three types of segmentation techniques based on the degree of automation in the process. Manual segmentation consists of manual delimitation of the boundaries of the area of interest, using the interface of a software tool. Semiautomatic segmentation uses software algorithms that process initial data provided manually and creates a label with the possibility of manual editing of the result. Automatic segmentation does not need any information to be provided manually and creates labels based only on the image provided, with the possible inclusion of predetermined parameters and prior training. Although automatic segmentation seems to be the most attractive option, mainly because of time savings, it demands high computational resources and is not an easy task in general, especially in cases of heterogeneous tumors (6). Segmentation is an essential step in radiomics, but it is important that segmentation is accurate and reproducible (7).

The purpose of this study is to evaluate the reproducibility of manual and semiautomatic segmentation of osteosarcoma and Ewing sarcoma in MRI, and to evaluate if there are differences in the time required for segmentation using these methods. To the best of our knowledge, this information is not available in the medical literature.

## Material and Methods

The study was approved by the Institutional Review Board (IRB) on Research Ethics (Faculdade de Medicina de Ribeirão Preto, Universidade de São Paulo). The IRB waived the requirement of informed consent. All patient information contained in the MRI DICOM files was anonymized using KPACS software (IMAGE Information Systems Ltd., Germany). To ensure patient privacy, the patients were identified through numbers (e.g., Patient #1, Patient #2) and not by name or initials.

### Image and case selection

Cases from January 2006 to August 2016 were selected from our institution's (FMRP-USP) Radiology Information System (RIS) by searching the keywords “osteosarcoma” or “Ewing sarcoma” in the conclusion of MRI reports. We included only cases for which diagnosis was confirmed by histopathology reports available in our institution's Hospital Information System (HIS).

All MRI data were acquired with Philips Achieva 1.5 Tesla MRI system (Philips Medical Systems, The Netherlands). MRI images with T1 weighting (T1WI) with fat suppression after intravenous administration of gadolinium (T1WI FS GD) were used to segment bone sarcomas in the axial plane. T1WI without fat suppression images, prior to gadolinium administration, were also available in the axial plane to assist in the process of segmentation in the T1WI FS GD images. The mean repetition time (TR) was 523 ms (ranging from 352 to 663 ms) and the mean time of echo (TE) of T1WI was 14 ms (ranging from 10 to 37 ms). The mean TR and TE in T1WI FS GD were, respectively, 523 ms (ranging from 352 to 663 ms) and 14 ms (ranging from 10 to 37 ms). The mean field-of-view (FOV) was 392×392 pixels (ranging from 240×240 to 640×640 pixels), with mean spatial resolution of 0.679 mm/pixel (ranging from 0.223 to 1.258 mm/pixel), and the mean slice thickness was 5.6 mm (ranging from 3.0 to 8.0 mm) for both sequences.

The inclusion criteria were as follows: osteosarcomas and Ewing sarcomas confirmed by histopathology analysis, MRI acquired prior to any invasive diagnostic procedure or any therapeutic intervention, and tumor site at the appendicular skeleton or pelvic bones. The exclusion criteria were cases acquired at 3.0 Tesla MRI, MRI acquired after any invasive diagnostic procedure or any therapeutic intervention, and MRI studies without acquisition of both axial T1WI and T1WI FS GD.

**Figure 1:**
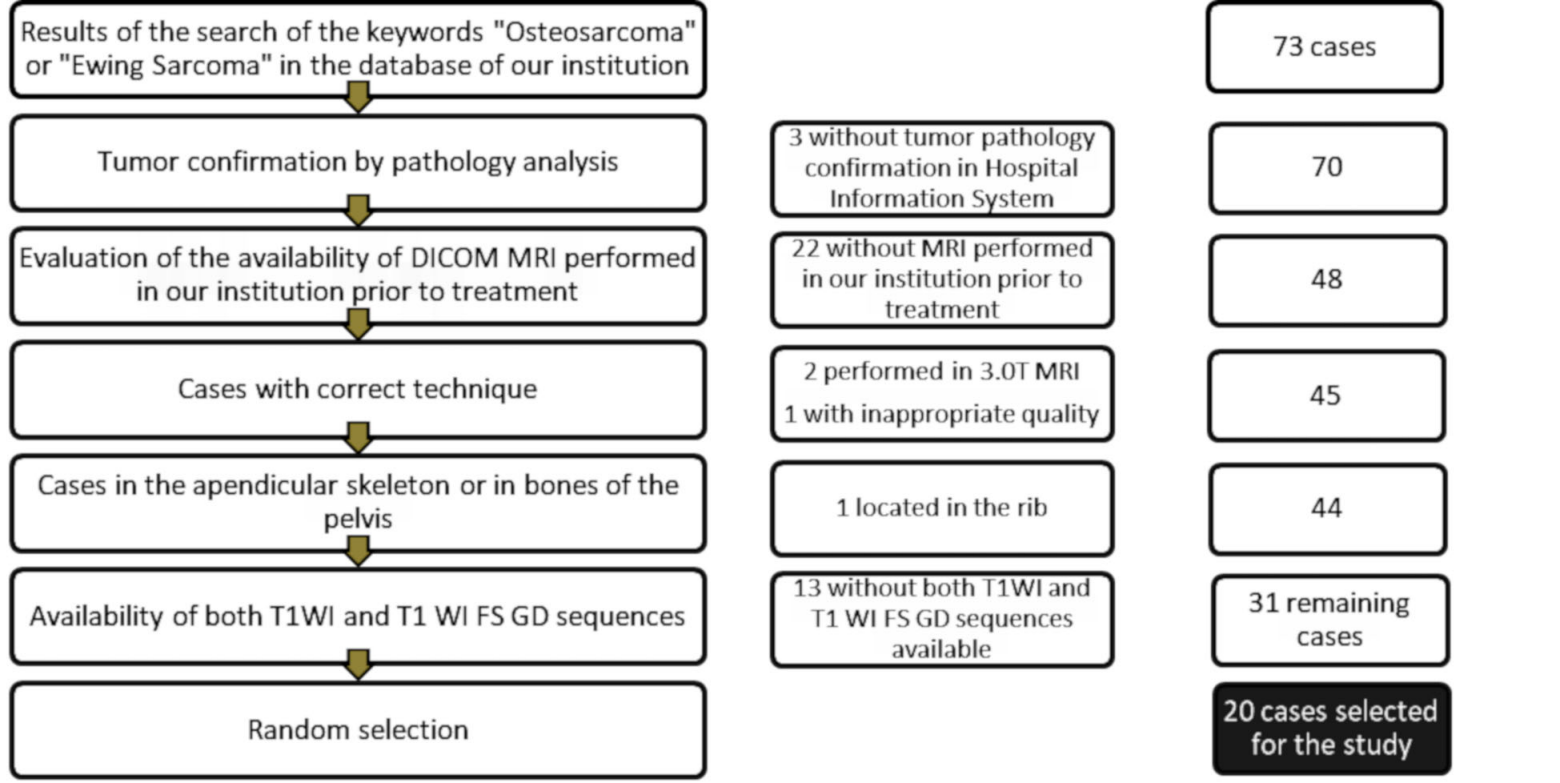
Flowchart of study sample selection.

Figure 1 summarizes the sampling steps. Twenty patients were randomly selected from the available cases. The software available in the internet site <https://www.randomizer.org/> was used for this task. The demographic characteristics of this sample were collected from our institution's HIS: mean age of 14 years (ranging from 2 to 35 years), six females and fourteen males, twelve osteosarcoma cases (representing 60% of the total cases), and eight cases of Ewing sarcoma. The complete demographic data collected are reported in Table 1.

**Table 1:** Distribution of cases according to gender, age, location, and histopathological result of the tumor.

Case	Gender	Age (years)	Location in skeleton	Tumor
1	F	8	Right femur	Osteosarcoma
2	M	35	Left calcaneus	Osteosarcoma
3	M	11	Right tibia	Ewing sarcoma
4	M	9	Right fibula	Ewing sarcoma
5	M	9	Left iliac	Ewing sarcoma
6	M	20	Right femur	Osteosarcoma
7	M	13	Right femur	Osteosarcoma
8	F	15	Right ischiopubic ramus	Ewing sarcoma
9	M	12	Right femur	Osteosarcoma
10	F	11	Right femur	Osteosarcoma
11	M	21	Left iliac	Ewing sarcoma
12	M	10	Right humerus	Ewing sarcoma
13	F	11	Right fibula	Ewing sarcoma
14	M	21	Right femur	Osteosarcoma
15	F	17	Right humerus	Osteosarcoma
16	F	20	Right femur	Osteosarcoma
17	M	16	Left tibia	Osteosarcoma
18	M	2	Right ulna	Ewing sarcoma
19	M	19	Right radius	Osteosarcoma
20	M	4	Right femur	Osteosarcoma

F: female; M: male.

### MRI analysis and segmentation

Three radiologists manually segmented the bone sarcomas on the MRI studies; two of them (Observers 1 and 2: O1 and O2) were in musculoskeletal radiology fellowship training, while Observer 3 (O3) was a staff radiologist with five years of experience in musculoskeletal radiology. The observers were not made aware of the results of radiology or pathology reports, clinical data, or the results of segmentation of the other observers. One month after initial segmentation, O1 performed a second manual segmentation task, and after one more month, O1 performed semiautomatic segmentation of the cases.

**Figure 2:**
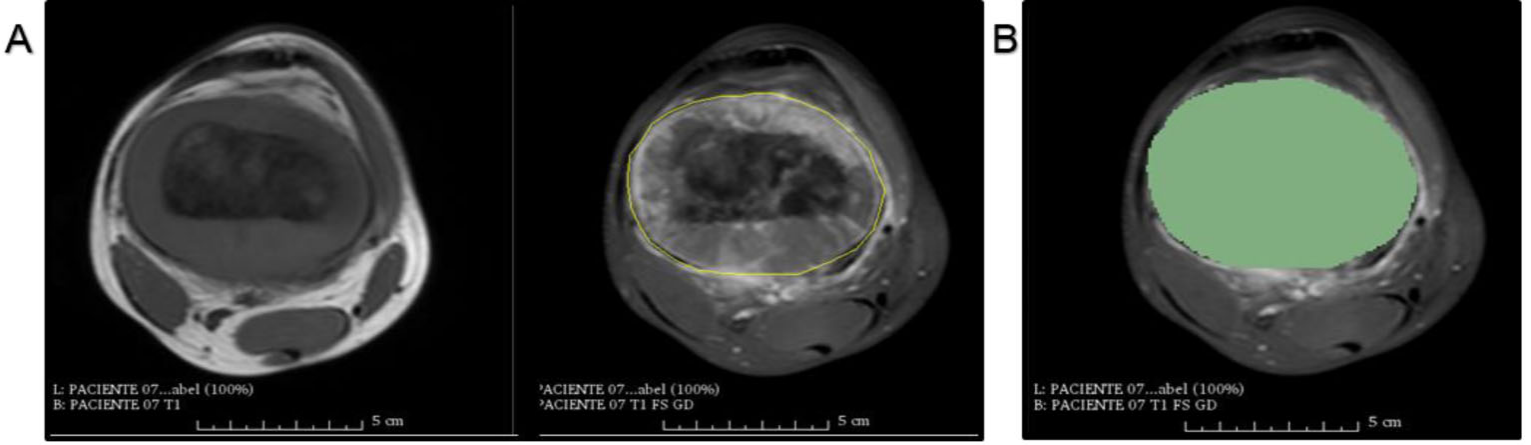
Slicer's GrowCut manual segmentation on axial plane using femoral osteosarcoma magnetic resonance imaging. **A**, T1 sequence image is on the left and T1 FS GD sequence image is on the right. Manual segmentation of the tumor in axial T1 FS GD images demarcated by yellow line. **B**, Same axial T1 FS GD image of (**A**), after marking the segmented area or “label” by the software (in green). T1WI: T1 weighting; T1WI FS GD: T1 weighting with fat suppression after intravenous administration of gadolinium.

The image segmentation tasks were performed using 3D-Slicer software version 4.6.2. This is an open-source software platform for medical image processing available at https://www.slicer.org. The GrowCut tool (available in 3D-Slicer software) provides an algorithm to perform semiautomatic segmentation. This software was validated and used in previous studies that segmented different types of neoplasms in MRI (8–10). Figure 2 shows the interface of the software after manual segmentation of a tumor.

Segmentation was performed on axial plane images, and for both manual and semiautomatic segmentation, the most cranial slice and the most caudal slice including the tumor were excluded to avoid partial volume artifacts. Manual segmentation was performed by drawing the boundaries of the sarcoma slice-by-slice. The time required for this process (the interval from opening the image archive to the moment when the segmentation was finalized) was measured for O1 and O3.

**Figure 3:**
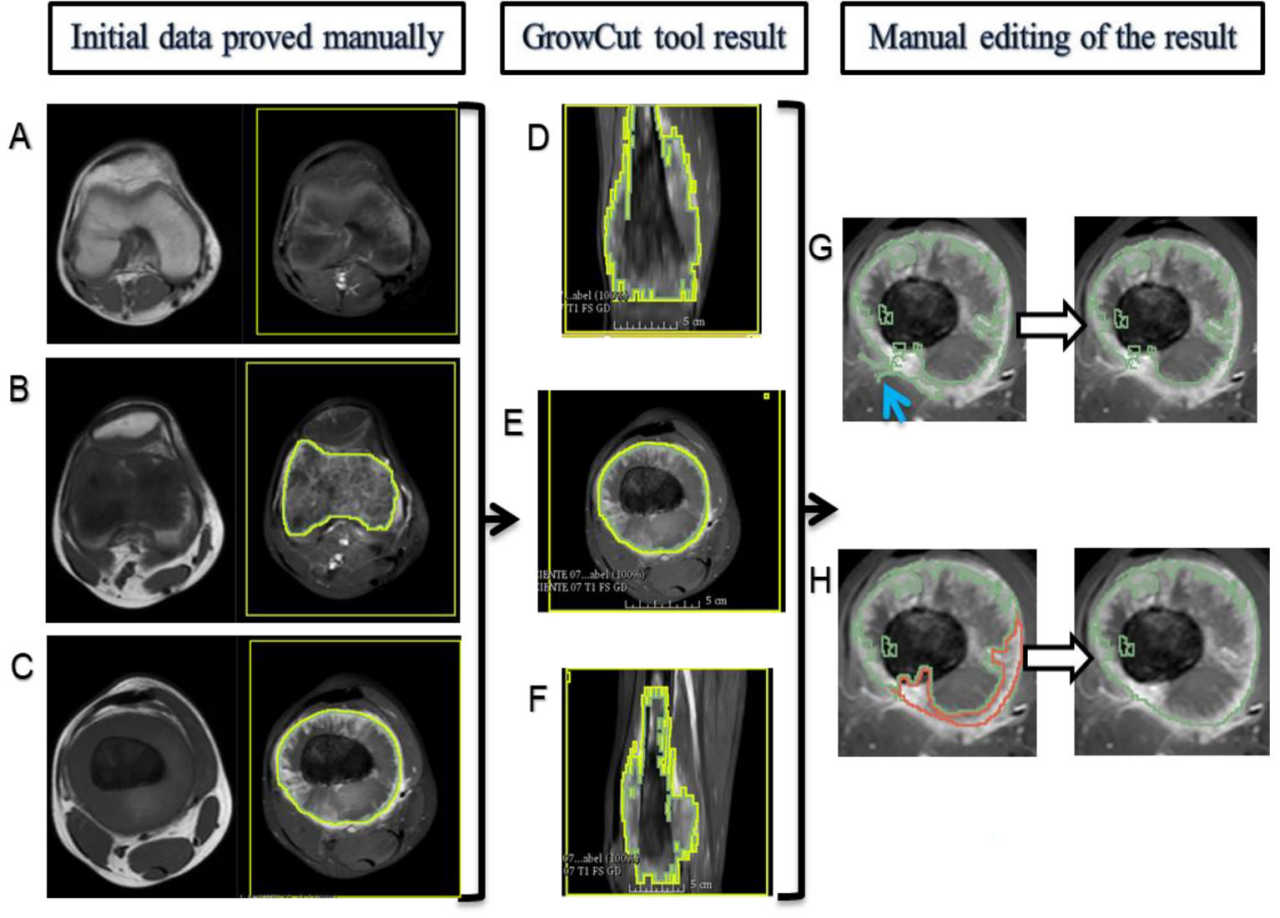
3D-Slicer's GrowCut semiautomatic segmentation steps of a femoral osteosarcoma magnetic resonance imaging case. In the first column (**A**, **B**, and **C**), the T1WI sequence images are on the left and the T1WI FS GD sequence images are on the right. In the second (**D**, **E**, and **F**) and third columns (**G** and **H**), there are only T1WI FS GD sequence images. **A**, Yellow rectangular area of background tissue beyond the inferior margin of tumor. **B** and **C**, Area of interest from the tumor tissue within green mark and background tissue within yellow mark in the extremities of the tumor (**B**) and in the middle portion of the tumor on the longitudinal axis (**C**). **D**, **E**, and **F**, Segmented volume of interest within green mark after GrowCut tool processing in coronal (**D**), axial (**E**), and sagittal (**F**) planes. **G** and **H**, Manual editing of GrowCut tool excluding areas from the volume of interest (within green mark) that do not contain tumor tissue, as indicated with the blue arrow in (**G**), and including areas with tumor tissue in the volume of interest, as shown in area marked in red (**H**).

Semiautomatic segmentation was performed by drawing the lesion boundaries and identifying all other areas outside the tumor as not belonging to the tumor (background). In this method, first manual segmentation was made in the extreme slices that would be included in the tumor as well as a slice in the middle of the tumor. After that, the GrowCut tool processed the data provided and the final result was a segmented volume. The segmented volume obtained in the previous step was submitted for manual editing by the same observer to exclude areas segmented by the tool that were deemed not to belong to the sarcoma and to include areas that were considered to belong to the tumor but were not segmented by the tool. Figure 3 summarizes the semiautomatic segmentation steps. The time required for semiautomatic segmentation was also measured.

The sarcoma tissue was recognized as low-T1WI-signal tissue and by contrast enhancement on T1WI FS GD, both in intra- and extra-osseous compartments. Cortical bone was included in the result of segmentation when there was adjacent extra-osseous mass, evident discontinuity of the cortical bone, or if the intramedullary component of the sarcoma reached the internal cortical layer and enhancement of the periosteum was present. Tendon and neurovascular bundles were included in the segmented area if they were completely contained by the tumoral tissue. Skip lesions and articular recess effusion or synovitis were not included in the segmented regions.

### Similarity between segmented regions

The dice similarity coefficient (DSC) and Hausdorff distance (HD) were used to evaluate similarity between results of segmentation. DSC measures spatial overlap and is a metric for validation of reproducibility; it is commonly used for validation of volumetric segmentation in medical images (11). The absolute value of DSC is difficult to interpret, but according to Zijdenbos et al. (12) and Bartko (13), DSC greater than or equal to 0.7 indicates excellent agreement between two segmented regions. Based on analysis of the kappa statistic, a DSC result between 0.61 and 0.80 is considered substantial, and one between 0.81 and 1.00 is considered almost perfect or excellent (14). The DSC between two A and B sets, representing the results of two segmentation methods in the present study, is defined as

$$\mathrm{DSC}=\frac{2|A\cap B|}{\left|A|+|B\right|}$$

HD is derived by computing the minimum distance from each point in A to all points in B, and then taking the maximum of such values over all points in A. Since this measure is not symmetric, the largest value computed as above from A to B and from B to A is taken as the more general value of HD. HD is a parameter that reflects the worst situation in assessing the similarity of two regions, because it indicates the maximum discrepancy between the two contours, even if the discrepancy occurs locally in a small portion of the segmented regions. Lower HD values represent smaller distances between the points on the contours of two results of segmentation being compared, and therefore, the more similar the results, the closer to zero is the value of HD.

### Statistical analysis

Statistical analysis was performed for DSC, HD, tumor volumetry, and segmentation time. The similarity of regions obtained from manual and semiautomatic segmentation techniques was assessed using DSC and HD. A professional statistician tested the distributions and applied the statistical tests as adequate for each case. The *t*-test for independent samples and Tukey’s multiple comparison tests were performed to compare DSC and HD. Mann-Whitney and Kruskal-Wallis multiple comparison tests were performed to compare the volumes of the segmented regions and segmentation times. Additionally, intraclass correlation was performed to compare measured tumor volumes. All statistical tests were performed using Minitab 17 (Minitab, LLC., USA) and MedCalc 14.8.1 (MedCalc Software, Belgium) software tools and a statistical significance level of 5% (P value ≤0.05) was defined for this study.

## Results

### Intraobserver agreement

The mean DSC was 0.91, with a standard deviation (SD) of ±0.03 (maximum: 0.97; minimum: 0.83). The mean HD was 11.74±6.41 mm (maximum: 28.73 mm, minimum: 3.37 mm). The mean volume of the first manual segmentation performed by O1 over all cases was 270.1 cm^3^ (±259.6 cm^3^) and mean volume of the second segmentation was 247.3 cm^3^ (±238.5 cm^3^). There was no statistically significant difference between the tumor volumes of the first and second segmentation exercises (P=0.881). The ratio between the tumor volumes of manual segmentation of the same cases by O1 was 1.01 (101%), with SD of ±39% (maximum: 270%, minimum: 76%). The intraclass correlation coefficient comparison between these volumes was 0.928 (with 95% confidence interval 0.827 to 0.970).

The average time for manual segmentation was 616.8±390.1 s (maximum: 1811 s, minimum: 175 s) in the first reading and 518.4±325.1 s (maximum: 1710 s, minimum: 237 s) in the second. There was no statistically significant difference when comparing the times for manual segmentation by O1 (P=0.280). Data of the manual segmentation exercises of O1 and the related statistical analysis are reported in Table 2.

**Table 2:** Dice similarity coefficient (DICE) and Hausdorff distance (HD) descriptive statistics for manual segmentation by Observer 1.

Manual segmentation	Variables	
	DICE	HD max (mm)
First *vs* second O1 segmentation		
Mean±SD	0.91±0.03	11.74±6.41
Median	0.93	9.77
CV (%)	4.12	54.54
Min;Max	(0.83; 0.97)	(3.37; 28.73)
95%CI	(0.90; 0.93)	(8.74; 14.74)

O1: observer 1; CV: coefficient of variation; CI: confidence interval.

### Interobserver agreement

In the comparison between O1 and O2, the mean DSC was 0.90±0.05 (maximum: 0.96; minimum: 0.73); the mean HD was 14.01±7.18 mm (maximum: 33.40 mm, minimum: 4.67 mm). In the comparison between O1 and O3, the mean DSC was 0.89±0.03 (maximum: 0.97; minimum: 0.84); the mean HD was 14.37±5.49 mm (maximum: 26.83 mm, minimum: 3.93 mm). There was no statistically significant difference in relation to the DSC and HD obtained in the comparison of O1 *vs* O2 with those obtained in the comparison of O1 *vs* O3 (P value of 0.606 and 0.863, respectively).

In the comparison between O2 and O3, the mean DSC was 0.86±0.05 (maximum: 0.96, minimum: 0.75); the mean HD was 7.4±9.18 mm (maximum: 36.00 mm, minimum: 6.16 mm). There was no statistically significant difference in relation to the DSC and HD obtained in the comparison of the O1 *vs* O2 with those obtained in the comparison of O2 *vs* O3 (P value of 0.609 and 0.167, respectively), and also in relation to the DSC and HD for O1 *vs* O3 and O2 *vs* O3 (P value of 0.633 and 0.840, respectively).

The mean volume was 247.3±238.5 cm^3^ for O1, 249.5±230.8 cm^3^ for O2, and 265.3±250.3 cm^3^ for O3. There was no statistically significant difference between the volumes for the three observers (P>0.05). The intraclass correlation coefficient for the comparison of the volume for O1 *vs* O2 was 0.996 (95%CI 0.991 to 0.998); O1 *vs* O3 was 0.993 (95%CI 0.983 to 0.997); and O2 *vs* O3 was 0.992 (95%CI 0.981 to 0.997).

The segmentation time was compared only between O1 and O3. The mean time for O1 was 518.4±325.1 s and for O3 was 634.9±299.2 s (P=0.163).

### Semiautomatic segmentation

The mean DSC was 0.88 (±0.05, maximum: 0.96, minimum: 0.74) and the mean HD was 12.46 mm (4.95 mm, maximum: 22.01 mm, minimum: 5.38 mm) in the comparison between semiautomatic and manual segmentation. Table 3 gives the data for DSC and HD in the comparison between manual and semiautomatic segmentation.

**Table 3:** Dice similarity coefficient (DICE) and Hausdorff distance (HD) descriptive statistics for comparison between results of manual and semiautomatic segmentation.

Segmentation	Variables	
	DICE	HD max (mm)
Manual *vs* semiautomatic O1 segmentation		
Mean±SD	0.88±0.05	12.46±4.95
Median	0.90	12.31
CV (%)	5.7	39.7
Min;Max	(0.74; 0.96)	(5.38; 22.01)
95%CI	(0.86; 0.90)	(10.14; 14.78)

O1: observer 1; CV: coefficient of variation; CI: confidence interval.

The mean tumor volume was 247.3±232.5 cm^3^ (maximum: 1044.6 cm^3^, minimum: 10.1 cm^3^) for manual segmentation and 251.4±240.8 cm^3^ (maximum: 1047.6 cm^3^, minimum: 15.3 cm^3^) for semiautomatic segmentation (P=0.946).

The segmentation time was different between the results of manual and semiautomatic segmentation (P=0.05). In manual segmentation, the mean time was 518.4±325.1 s (maximum: 1710 s, minimum: 237 s). In semiautomatic segmentation, the mean segmentation time was 325.8±124.0 s (maximum: 745 s, minimum: 166 s).

### Comparison of segmentation of Ewing sarcoma and osteosarcoma

When analyzed separately, the results of intraobserver comparison of segmentation of osteosarcoma were DSC of 0.92±0.03, mean HD of 11.20±5.93 mm, and mean volume of 231.4±185.4 cm^3^ for the first segmentation and 224.4±167cm^3^ for the second segmentation. In the intraobserver comparison, the results for Ewing sarcoma cases were mean DSC of 0.90±0.002, mean HD of 12.53±7.40 mm, and mean volume of 278.2±323.6 cm^3^ for the first segmentation and 281.7±328.9 cm^3^ for the second segmentation. There was no statistically significant difference between the mean volumes of osteosarcoma and Ewing sarcoma in the first and the second manual segmentation (P>0.05).

When the tumors were analyzed separately in interobserver comparisons, the results were mean DSC ≥0.89 and mean HD ≤15.48 mm (P>0.05)

When the tumors were analyzed separately in the comparison of manual and semiautomatic segmentation, the results were mean DSC ≥0.88 and mean HD ≤12.61 mm (P>0.05).

## Discussion

To the best of our knowledge, there are no studies published in the literature that have compared manual and semiautomatic segmentation of bone sarcomas in MRI or validated semiautomatic segmentation of osteosarcoma and Ewing sarcoma. Studies involving the segmentation of other solid tumors, such as non-small-cell lung carcinoma (10) and multiform glioblastomas (9), have obtained good results in the evaluation of intraobserver reproducibility, using both DSC and HD for comparison.

In this study, we used MRI as the imaging modality for segmentation of bone sarcomas. MRI is considered the method of choice for evaluating intra- and extra-osseous tumor extension in bone sarcomas, and for the assessment of infiltration of adjacent tissues, such as muscles and neurovascular bundles. MRI is the reference standard for the evaluation of tumor components, such as cysts, adipose tissue, fibrous tissue, chondroid tissue, calcifications with some limitation of sensitivity, and tissue necrosis (15,16); the representation of such components may be useful for extraction of data in the context of radiomics.

Three MRI sequences are commonly used in clinical practice for cases of bone sarcoma: T1WI, T2WI with fat suppression, and T1WI FS GD. For segmentation, it was necessary to choose a sequence to delimit the tumor. According to Baweja et al. (17) in a study that correlated MRI with surgical findings in bone tumors, bone marrow involvement is best evaluated in the T1 sequence in the coronal or sagittal plane, the involvement of soft tissues is best evaluated in the T2 sequence in the axial plane, cortical involvement is best evaluated in the longitudinal or axial plane with T1, and neurovascular involvement is best evaluated in the T2 or T1WI FS GD sequence in the axial plane. The T1WI FS GD sequence was chosen in this study to perform segmentation because this sequence shows good tissue contrast between the tumor and non-tumoral tissues, both in the intra- and extra-osseous compartments. Post-contrast MRI is adequate for the definition of cystic lesions, the pattern of vascularization of a tumor, and to confirm necrotic areas. Additionally, the T1WI FS GD sequence has been used in radiomics in previous studies (3,18 [Bibr B19]).

The non-fat-suppressed T1WI sequence was used as an auxiliary tool in segmentation because this sequence shows excellent contrast between the intraosseous tumor and preserved bone marrow. However, since tumor tissue and the surrounding soft-tissue structures both show low signal in the T1WI sequence, it can be difficult to delimit the tumor. Another useful contribution of the non-fat-suppressed T1WI sequence is to evidence the existence of adipose tissue permeating areas of edema, allowing differentiation between edema and tumor solid tissue in extra-osseous compartments (3,15,18–20). T2WI sequences usually show high signal in tumor tissue and perilesional edema, and the interface between these areas may be obscure; this could cause a tendency to overestimate the tumor dimensions in T2WI (15,18). We chose the axial plane for segmentation because this plane was available in all MRI acquisitions to study bone sarcomas and regional anatomical relations are well depicted in this plane. During segmentation, the most cranial and the most caudal slices of the tumor volume were not used. Despite the loss of these two slices of tumor volume, we standardized this procedure to avoid the potential inclusion of non-tumoral tissue in the volume of interest, due to partial volume effect.

We included cortical bone in tumor segmentation when there were signs of cortical tissue rupture or resorption, in cases of soft tissue components external and internal to the cortical bone, and in cases in which the medullary component of the tumor reached the internal cortical layer and simultaneously there was post-contrast periosteal enhancement. Kumar and Hari (21) reported that visualization of cortical rupture on T1WI MRI was associated with involvement of the cortical bone in the tumor's histology in 90% of the cases. On the opposite side, when there was no cortical rupture detected by MRI, only 10% of the cases had histopathological involvement of the cortical bone by tumor cells. According to Zimmer et al. (22), cortical involvement is characterized by increased cortical signal in MR sequences with loss of bone marrow or soft tissue interface adjacent to cortical bone. Our criteria for segmentation are in agreement with these observations. While MRI is accepted as the most sensitive technique to detect bone marrow abnormalities, the use of MRI to assess cortical bone and periosteum abnormalities is less recognized but has been addressed in the literature (23–27).

Although some studies have identified tumor cells in peritumoral edema, soft tissue sarcomas (28), and glioblastoma (29), in this study, we chose to exclude peritumoral edema in the segmentation procedure. This option is related to the poor conspicuity of the edema that could potentially decrease the reliability of segmentation. Previous studies used the same strategy for segmentation of soft-tissue sarcomas and osteosarcoma xenotransplantation in murine models (30,31), in which areas of peritumoral edema were not segmented. Peritumoral edema may potentially contain useful information to be extracted and analyzed, but it would probably be better to evaluate this separately in relation to the solid tumor. We encourage future studies to verify this hypothesis.

Intraobserver reproducibility of segmentation was excellent. We found no significant difference in DSC and HD in interobserver analysis. There was also excellent interobserver agreement, with high similarity indicated by DSC higher than 0.7 and close to 1. HD values obtained from interobserver comparisons were not significantly different compared with HD obtained from intraobserver analysis.

There was no significant difference between the volumes of repeated manual segmentation by the same observer. Additionally, the intraclass correlation coefficient was close to 1, indicating excellent intraobserver reproducibility in manual segmentation.

Interobserver comparison of the volumes of bone sarcomas showed no significant difference. Correlation coefficient values of the volume comparisons were close to 1. In addition, there was no significant difference in the evaluation of the volume of bone sarcomas when the observers were evaluated in pairs. This result indicates high similarity between the segmentation results of the observers.

The difference in time required by O1 for the two manual segmentation tasks performed was not significant. Segmentation time showed no significant difference between the observers.

The results indicated excellent agreement with high similarity between manual and semiautomatic segmentation, with DSC values higher than 0.75. The HD values obtained were similar to the values observed in the analysis of intraobserver and interobserver reproducibility, indicating that, in relation to these parameters, the reproducibility was similar. There was no significant difference between the volume of bone sarcomas when manual and semiautomatic segmentation were compared.

The time required for semiautomatic segmentation was significantly lower than the manual segmentation time. However, as the P value was in the limit of significance (P=0.05), more robust studies may be needed to reinforce our results. Other studies that analyzed segmentation of solid tumors have also found good similarity in manual and semiautomatic segmentations and observed a reduction in time with semiautomatic segmentation (8–10).

When compared separately, the results of segmentation of osteosarcoma and Ewing sarcoma were also excellent in intraobserver and interobserver agreement and without significant differences between the volumes of segmentation.

Our results have potential application to future research methodology using radiomic features in the evaluation of osteosarcoma and Ewing sarcoma. Previous studies have demonstrated the applicability of radiomics in the assessment of several types of tumors (32–37). In cases of bone sarcomas, research in radiomics is still scarce. Foroutan et al. (31) studied a model of osteosarcoma xenograft in mice and response after MK1175 and gemcitabine therapy. The authors performed manual segmentation of the tumors on MRI, and quantified volumetric variation and variation of distribution in ADC maps, demonstrating a correlation between the increase in signal in ADC maps and cellular apoptosis, in correlation with histopathological results. Wu et al. (38) studied manually segmented high-grade osteosarcomas in computed tomography images and analyzed their characteristics in the context of radiomics. They found that a radiomic nomogram, which combines clinical and radiomic features, was better to predict survival when compared with only clinical factors.

There are limitations in the present research. The study was retrospective and the number of cases was relatively small. Only a single observer used the GrowCut tool for semi-automated segmentation and this was the only observer that measured segmentation time for comparison of different segmentation techniques. Another limitation is the fact that, in the interobserver comparison, only two of the three observers measured the segmentation time. Recently, deep learning and artificial intelligence strategies have gained attention in the scenario of research in medical imaging and oncology (39). Deep learning algorithms do not need segmentation, but this approach requires a large database for training, which is difficult to obtain in the case of uncommon neoplasms such as bone sarcomas.

Our results showed excellent intra- and interobserver agreement for manual segmentation of osteosarcoma and Ewing sarcoma. In addition, there was high similarity between the results of manual and semiautomatic segmentation, with a significant reduction of segmentation time with the semiautomatic method.
